# Regulating Internet Trade in CITES Species

**DOI:** 10.1111/cobi.12019

**Published:** 2013-02-08

**Authors:** Maurizio Sajeva, Claudio Augugliaro, Matthew J Smith, Elisabetta Oddo

**Affiliations:** * Dipartimento di Biologia Ambientale e Biodiversità, Università di Palermovia Archirafi 18,, 90123, Palermo, Italy; † Computational Science Laboratory, Microsoft Research7 J J Thompson Avenue, Cambridge,, CB3 0FB, United Kingdom

International trade in species that are or may be endangered by collection from the wild is regulated under the Convention on International Trade in Endangered Species of wild fauna and flora (CITES) for 176 member States (Parties). Internet commerce is a relatively new route for such trade. In 2007, the CITES Secretariat asked Parties to collect information on internet wildlife trade and report problems and implemented regulations. The reports indicated it was difficult to even approximate the influence of e-commerce on CITES-listed species (CITES Secretariat 2009). We report a case study in which we quantified international transactions over an internet auction site of CITES-listed cacti and cross-checked them with CITES trade data. Our results were both surprising and alarming.

Species protected under CITES are included in different CITES appendices according to their level of threat of extinction from international trade. Taxa listed in Appendix I are endangered and wild collection could cause their extinction, and taxa listed in Appendix II either may become endangered if wild collection is not regulated or are similar in appearance to other species listed in Appendix I or II (see http://www.cites.org for full definitions). The treaty operates through the issue and control of permits that apply to regular and internet trade. Trade in wild specimens is either prohibited (species listed in Appendix I) or regulated by permits (species listed in Appendix II), although some Parties implement stricter regulations. Export permits must also be issued for certified artificially propagated plants. International trade of plants listed in Appendix I is allowed only if they are artificially propagated. Plants listed in Appendix I that are artificially propagated for commercial purposes are included in Appendix II (Article VII, paragraph 4 of the Convention), and the exporting enterprises should be registered in accordance with Resolution of the Conference of the Parties 9.19 (Revised at CoP15).

Parties must submit annual reports to the CITES Secretariat listing the number and type of permits and certificates granted, the States with which such trade occurred, and the quantities and types of specimens traded. Some Parties report actual trade, whereas others report the permits issued. Trade data from these reports are stored in the CITES Trade Database (CTD) (http://www.unep-wcmc-apps.org/citestrade/). We used this information to assess the implementation of the Convention for international trade in CITES species over the internet. We investigated e-commerce in 2010 for cacti as a case study. All members of the Cactaceae, with the exception of 3 genera (*Pereskia, Pereskiopsis*, and *Quiabentia*) are listed in either Appendix I or II. We also restricted our analyses to species listed in Appendix I because an export permit is mandatory to export and import live plants.

We monitored buyer–seller interactions on an internet auction site (not identified here) and recorded sales of live plants that were successfully completed and for which we could identify the plant's country of origin and its destination. We compared this information with data on export permits for live plants in the CTD. Although these report trade data, rather than individual permits, they should reflect the internet trade if export permits were applied for, as required for all such transactions involving CITES Parties (the case for all transactions we recorded). Trade within the European Union does not require CITES permits, so we excluded these transaction and sales to Parties that have submitted official reservations against being regulated by CITES for certain cacti. All relevant exporting Parties had submitted their 2010 reports by the time of our analyses.

We monitored 24 sellers over 6 months, twice weekly, until 1000 cacti listed in Appendix I had been sold. There were 978 such sales of a single plant, 1 sale with 6 plants, and 1 sale with 16 plants. We checked all scientific names and controlled for nomenclature differences between the names used in auctions and the official names used on CITES permits. Subspecies were noted if considered valid by the CITES cacti checklist. It is possible that the CTD records corresponding to a particular transaction actually corresponded to a different transaction with the same details. Our figures therefore represent the maximum number of transactions for which CITES export permits could have been issued. We did not check whether species were traded under permits spanning multiple years or under invalid permits, but we expected these would have only minor effects.

Our data set contained roughly a quarter of the cactus plants for which CITES permits were issued in 2010. There were large discrepancies in the number of plants for which permits were issued and the number of plants traded in online transactions (Table 1). Our results suggest that only 10% of the plants traded were even potentially legal. Major discrepancies were also apparent in the number of species and number of importing and exporting countries between the online auctions and permits issued for that year (Table 1).

**Table 1:** Sales of cacti listed on CITES Appendix I on an internet auction site and permits issued by CITES Parties in 2010

	Cacti auction-site sales	CITES trade reported^a^
Number of live plants	1000	3973
Number of live plants for	107	
which CITES permits matched a trade database entry	(10.7%)	
Number of species	54	29
Number of exporting countries	11	8
Number of importing countries	44	11

a On the basis of export permits issued by national CITES authorities.

We suspect that most transactions we recorded were of artificially propagated plants. The United States was the only Range State recorded as exporting native species (approximately 8% of the recorded transactions). Few of the cacti sold that were pictured on the website had visible characteristics that could plausibly be associated with the plant being of wild origin. We therefore expect the recorded transactions to have only minor effects, if any, on wild populations. Nonetheless, an export permit is mandatory to export and import these plants. Therefore, the potentially wide scale of the illegal global trade that our results suggest should raise concerns about the adequacy of the protection for CITES species. For example, wild populations of some cacti listed in Appendix I may number only a few dozen individuals in their natural habitat (Hernandez et al. 2010), for which collectors are willing to pay high prices (Robbins 2003). Internet auction sites should be monitored more widely to investigate trade in CITES species with the aim of more effectively regulating trade in rare plants.
